# Pharmacometabolomic signatures as individualised biochemical response evidence: towards a precision jurisprudence framework for forensic toxicology and criminal responsibility

**DOI:** 10.3389/fphar.2026.1881604

**Published:** 2026-10-01

**Authors:** Okechukwu Paul-Chima Ugwu, Maria Edet Umo, Richard Akpanke Akwagiobe, Akor Innocent Ondale

**Affiliations:** 1 Department of Research and Publication, Kampala International University, Kampala, Uganda; 2 Department of Law and Jurisprudence, School of Law, Kampala International University, Kampala, Uganda

**Keywords:** criminal liability, CYP450, forensic toxicology, individualised biochemical response evidence, metabolomics, pharmacogenomics, pharmacometabolomics, precision medicine

## Abstract

Precision medicine is transforming drug response from a population level assumption into an individualised biochemical event. Pharmacometabolomics, which profiles metabolic signatures associated with drug exposure and response, offers a mechanistic basis for explaining interindividual variability in therapeutic efficacy, adverse drug reactions, neurocognitive impairment and toxicological ambiguity. Legal systems, however, continue to evaluate drug related conduct largely through conventional toxicology, generalised impairment thresholds and binary assumptions about intent, voluntariness and negligence. This Perspective proposes individualised biochemical response evidence, described here metaphorically as an emerging category of biochemical legal identity, as a prospective research and governance framework rather than a validated evidentiary standard. Its primary focus is forensic toxicology and criminal responsibility, with medical negligence, product liability, AI guided prescribing, pharmaceutical regulation and biochemical privacy discussed as secondary implications requiring separate validation. The claim is not that metabolomic profiles are currently capable of establishing individual causation, voluntariness or capacity, but that a disciplined evidentiary pathway, moving from biological plausibility through analytical validity, clinical validity and forensic validity to legal admissibility, would be required before such evidence could inform these determinations. A precision jurisprudence framework is proposed to guide this transition while preserving safeguards against biochemical determinism, privacy intrusion and algorithmic opacity.

## Introduction

Drug response is increasingly understood as an individualised biochemical phenomenon rather than a uniform pharmacological effect, consistent with the broader transition toward personalised and precision medicine (Collins and Varmus, 2015; Hamburg and Collins, 2010). Variation in absorption, distribution, metabolism, excretion, receptor sensitivity, inflammatory status, microbiome composition, disease state and concomitant medication can substantially alter therapeutic and behavioural outcomes. Pharmacometabolomics captures this variability by identifying metabolic signatures associated with drug exposure and response, thereby extending the clinical logic of precision medicine into drug response phenotyping (Beger et al., 2016; Kaddurah-Daouk and Weinshilboum, 2015; Nicholson et al., 2012; Wishart, 2016). The field is particularly important because metabolomic phenotypes integrate genomic, environmental, dietary, microbial and inflammatory influences that are not captured by genotype alone (Relling and Evans, 2015; Whirl-Carrillo et al., 2021).

The legal implications of this shift are substantial. Courts, forensic laboratories, regulators, clinicians, insurers and pharmaceutical manufacturers increasingly encounter disputes involving medication associated behavioural change, toxicological uncertainty, adverse drug reactions, impaired judgement and AI supported prescribing (Cohen et al., 2014; Rajkomar et al., 2019). Yet many legal frameworks remain dependent on static drug concentration evidence, generalised assumptions about impairment and population level expectations of responsibility and foreseeability. This Perspective proposes that pharmacometabolomic signatures may, in time and subject to validation, be understood through the metaphor of biochemical legal identity, meaning individualised and dynamic biochemical response evidence rather than a claim of biological uniqueness or a means of personal identification. Unlike genomic identity, which is relatively stable, pharmacometabolomic identity is dynamic, exposure dependent and temporally situated. It describes how a particular body transforms and responds to a particular drug under particular physiological conditions, and the term is used descriptively rather than as an assertion that such signatures can identify or characterise a person in a legal or forensic sense.

## From drug detection to individualised biochemical response evidence

Traditional forensic toxicology asks whether a substance was present, at what concentration it was present and whether that concentration is consistent with exposure, toxicity or impairment. These questions remain indispensable, but they are incomplete. Similar drug concentrations may produce materially different behavioural or cognitive effects across individuals because of CYP450 polymorphisms, active metabolite accumulation, hepatic function, renal clearance, drug interactions, inflammation and coexisting disease. The interpretive gap between exposure and effect is precisely where pharmacometabolomic evidence may, subject to future validation, acquire forensic and legal relevance.

Metabolomics is already recognised as relevant to forensic toxicology and forensic medicine, including intoxication interpretation, biomarker discovery, *post mortem* biochemical analysis and estimation of *post mortem* interval (Castillo-Peinado and Luque de Castro, 2016; Dawidowska et al., 2021; Nielsen et al., 2016; Szeremeta et al., 2021). This existing forensic utility does not, however, establish that a validated metabolomic response can support individual behavioural attribution or criminal responsibility; the two questions are distinct, and evidence supporting the former should not be read as evidence supporting the latter. The proposed category of biochemical legal identity therefore reframes evidence as dynamic, individualised, probabilistic and contextual. It is dynamic because metabolic signatures change over time; individualised because the same drug can produce different biochemical consequences in different persons; probabilistic because the evidence, once validated, would support likelihood rather than certainty; and contextual because legal relevance depends on causation, timing, behaviour and admissibility. As used throughout this Perspective, the term refers to individualised biochemical response evidence and carries no claim of biological uniqueness or forensic identification comparable to genomic identity testing.

## Distinguishing pharmacogenomics, pharmacokinetics, conventional toxicology and pharmacometabolomics

The arguments developed in this Perspective depend on evidentiary categories that are frequently conflated in translational discussion and should be defined precisely before their legal implications are considered.

Pharmacogenomics examines inherited genetic variation, such as CYP2D6, CYP2C19 and CYP2B6 polymorphisms, that predicts an individual’s capacity to metabolise particular drugs. Genotype informed prescribing guidelines issued by the Clinical Pharmacogenetics Implementation Consortium establish pharmacogenomic actionability, meaning that a genotype is associated with a reasonably predictable difference in drug handling that justifies a dosing or drug selection recommendation (Bousman et al., 2023; Crews et al., 2021; Lee et al., 2022). This actionability is a statement about population level prescribing guidance; it does not, by itself, validate the use of pharmacometabolomic evidence to establish behavioural impairment in an individual case.

Pharmacokinetics and conventional toxicology measure the parent drug or a known metabolite in blood, urine or tissue to establish exposure, concentration and consistency with a toxic or therapeutic range. Measuring a parent drug or an established active metabolite for this purpose remains conventional toxicology or therapeutic drug monitoring rather than pharmacometabolomics, even where the same analytical platform is used.

Pharmacometabolomics, by contrast, profiles a broader set of endogenous and drug related metabolites to characterise an individualised biochemical response to drug exposure, rather than simply confirming that exposure occurred (Kaddurah-Daouk and Weinshilboum, 2015; Rattray and Kaddurah-Daouk, 2017). The proposed evidentiary pathway from drug exposure and biological response through analytical measurement and integrated evidence to forensic interpretation and normative legal or regulatory decision-making is summarised in Figure 1. A pharmacometabolomic signature in this Perspective may refer to a single informative metabolite, a drug to metabolite ratio, a defined endogenous metabolite panel, a longitudinal change in such a panel, or a multivariate classifier derived from these measurements. These categories require different degrees of analytical and clinical validation, and none of them should be treated as interchangeable with pharmacogenomic actionability, therapeutic drug monitoring, or conventional toxicological interpretation. Distinguishing these categories is a precondition for the legal analysis that follows, because the evidentiary weight appropriate to each differs substantially.

**FIGURE 1:**
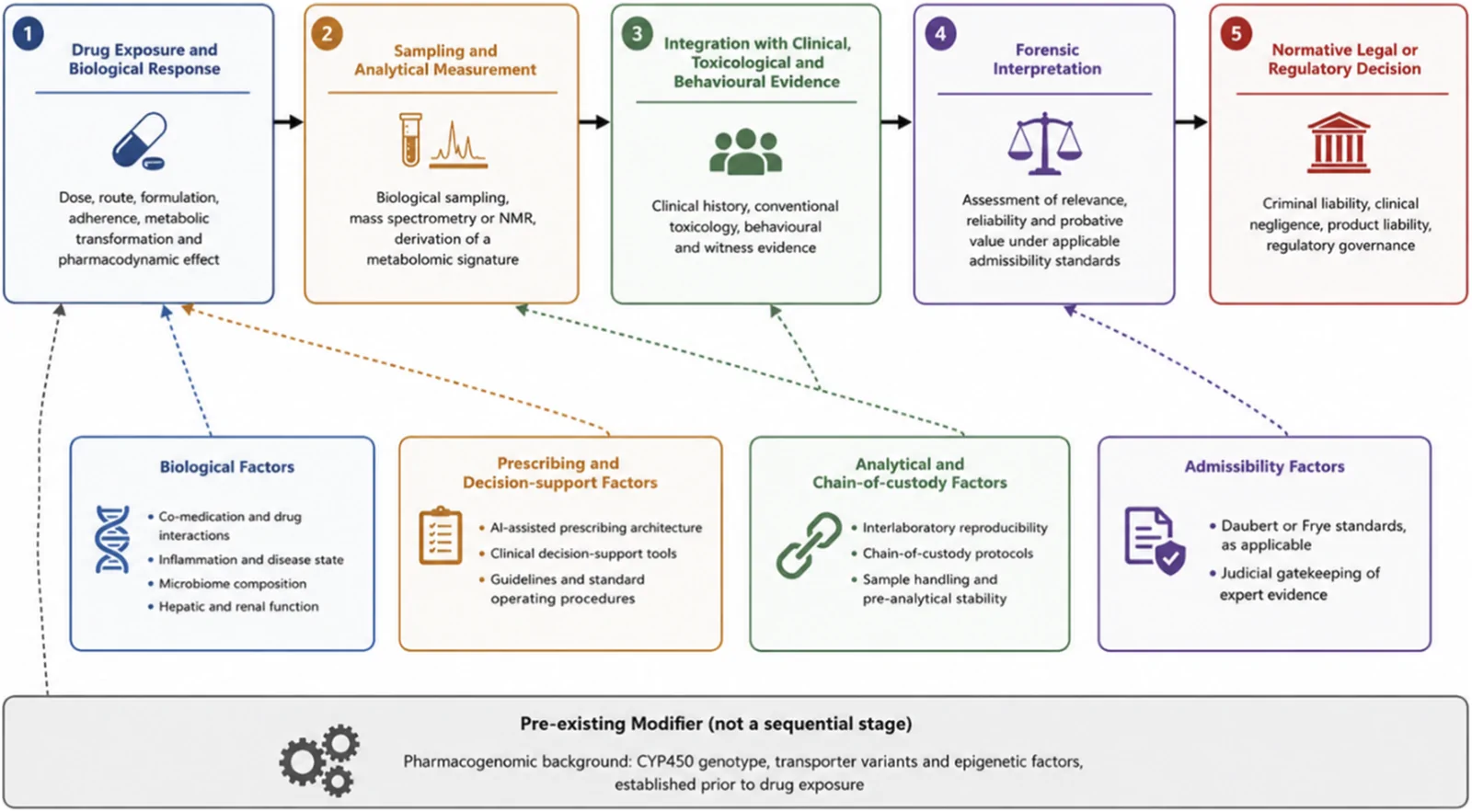
Conceptual framework. Drug exposure and biological response lead, through sampling and analytical measurement and integration with clinical, toxicological and behavioural evidence, to forensic interpretation and ultimately a normative legal or regulatory decision. Pharmacogenomic background (CYP450 genotype, transporter variants and epigenetic factors) is shown as a pre-existing modifier established prior to drug exposure rather than as a sequential stage, and informs Stage 1 only. Biological factors (co-medication and drug interactions, inflammation and disease state, microbiome composition, hepatic and renal function) and prescribing and decision-support factors (AI-assisted prescribing architecture, clinical decision-support tools, guidelines and standard operating procedures) primarily influence drug exposure and biological response and are therefore mapped to Stage 1 only. Analytical and chain-of-custody factors (interlaboratory reproducibility, chain-of-custody protocols, sample handling and pre-analytical stability) affect the sampling and analytical measurement process and are mapped to Stage 2 only. Admissibility factors (Daubert or Frye standards, as applicable, and judicial gatekeeping of expert evidence) operate at the level of forensic interpretation and are mapped to Stage 4 only; no modifying factor is shown as acting on Stage 5, which represents the normative legal or regulatory decision itself. Each modifying-factor group is therefore mapped to the single stage or stages it is understood to affect, rather than to all four preceding stages uniformly. NMR = nuclear magnetic resonance; CYP450 = cytochrome P450.

## Pharmacometabolomics and criminal responsibility

Criminal liability often turns on voluntariness, mens rea, knowledge, recklessness and capacity. Pharmacometabolomic evidence may, subject to the validation discussed throughout this Perspective, become relevant where drug exposure plausibly affects cognition, consciousness, behavioural inhibition or impulse control. Such evidence would be particularly relevant in disputes involving antidepressant associated behavioural change, opioid induced delirium, benzodiazepine associated automatism, stimulant associated psychosis, polypharmacy related cognitive impairment and unexpected neuropsychiatric adverse reactions.

The underlying pharmacological basis for interindividual variability in drug response is well established and is not in itself speculative. CYP2D6, CYP2C19, CYP2B6 and related enzymes influence the metabolism of several psychotropic and analgesic medicines, and Clinical Pharmacogenetics Implementation Consortium guidelines already provide genotype informed prescribing recommendations for serotonin reuptake inhibitor antidepressants and selected opioid therapies (Bousman et al., 2023; Crews et al., 2021). Pharmacogenomic implementation resources further show that drug response variability is being formalised through clinical annotation and evidence grading (Whirl-Carrillo et al., 2021). What remains unvalidated, and what this Perspective does not claim to have resolved, is whether a pharmacometabolomic signature can reliably identify drug related impairment in a specific individual, distinguish it from competing explanations such as pre-existing psychiatric illness, concurrent substance use or situational stress, or establish the causation, voluntariness or capacity that criminal responsibility requires. Until such validation exists, pharmacometabolomic evidence should be regarded as informing the biological plausibility of a claimed drug response rather than as proof of any legal element, and its introduction should always be accompanied by clinical, toxicological and behavioural evidence establishing the individual causal link that biological plausibility alone cannot provide.

## Foreseeability, negligence and an emerging duty of care

As pharmacogenomic and metabolomic tools become more clinically actionable, the standard of care may gradually evolve, although this evolution involves several distinct steps that should not be treated as a single continuous inference. Regulatory recognition of pharmacogenomic biomarkers in drug labelling demonstrates that drug response variability is no longer merely experimental; the United States Food and Drug Administration maintains a pharmacogenomic biomarker table identifying medicines whose labels contain relevant biomarker information (U.S. Food and Drug Administration, 2026). It is important to keep the underlying evidentiary category constant throughout this analysis: the FDA biomarker table cited here concerns pharmacogenomic biomarkers, that is, biomarkers derived from inherited genetic variation, and its regulatory recognition speaks to pharmacogenomic actionability rather than to pharmacometabolomic actionability. Consistent with the distinction drawn earlier in this Perspective, pharmacogenomic actionability should not be read as evidence of a corresponding level of regulatory recognition, clinical actionability or standard of care for pharmacometabolomic signatures specifically, and no equivalent regulatory recognition currently exists for pharmacometabolomic biomarkers as defined here. Regulatory recognition of this kind establishes that a biomarker exists and has been formally acknowledged. It does not, without further steps, establish clinical actionability in a given care setting, professional adoption of routine testing, an accepted standard of care, a breach of that standard, or causation between an omission and a resulting harm. Each of these is a separate evidentiary and legal question, and negligence analysis should keep them distinct rather than treating labelling recognition as if it were itself proof of a duty to test.

This nonetheless raises a Frontier negligence question worth articulating carefully: when might a failure to consider biochemical drug response risk become a breach of duty, once the intervening steps described above are satisfied? In clinical practice, the issue could arise where a prescriber disregards a genuinely known and clinically actionable pharmacogenomic risk, fails to monitor high risk polypharmacy, or relies on an algorithmic prescribing recommendation without evaluating patient specific metabolic vulnerability that the relevant professional community could reasonably have been expected to know about at the time of care. In product liability, an analogous question could arise where manufacturers fail to warn identifiable metabolic subgroups about foreseeable adverse reactions supported by adequate evidence. A mature precision medicine environment may eventually support an individualised duty of care in which biochemical variability is treated as a foreseeable dimension of safe prescribing, but this possibility is presented here as a prospective development contingent on the validation, professional adoption and standard setting discussed throughout, rather than as a present legal standard.

## Evidentiary safeguards and admissibility

The admissibility analysis in this Perspective is grounded primarily in United States evidentiary doctrine, reflecting the jurisdictions in which Daubert and Frye operate, and should be read with that scope in mind rather than as a universal statement about legal systems generally. In jurisdictions applying Daubert, scientific evidence must be grounded in scientific validity and must assist the trier of fact in resolving the issue before the court (Daubert, v. Merrell dow pharmaceuticals, 1993). In jurisdictions applying Frye, admissibility still turns substantially on general acceptance within the relevant scientific community (Frye, 1923). Other common law and civil law jurisdictions apply their own tests for expert evidence, voluntariness, mens rea, negligence and causation, and a full comparative analysis of those frameworks is beyond the scope of this Perspective; readers in other jurisdictions should treat the arguments here as illustrative of the underlying scientific and evidentiary issues rather than as a direct statement of their own law.

Within the United States framework, a legally credible pathway would require validated analytical methods, reproducible biomarker panels, clear temporal linkage between drug exposure and behaviour, integration with conventional toxicology and clinical history, transparent statistical interpretation, independent expert review and safeguards against deterministic overclaiming. No metabolomic signature should be treated as proof that a person lacked agency. Rather, it should be interpreted as one component within a wider evidentiary matrix that includes clinical records, toxicological findings, pharmacological plausibility, witness evidence, behavioural chronology and the applicable legal test. Scientific reliability, which concerns whether a method or signature is analytically and clinically sound, should also be kept conceptually separate from the subsequent and distinct normative judgement concerning legal responsibility, which remains for courts and juries to make.

## AI guided prescribing as a secondary application

AI guided prescribing systems increasingly promise dose optimisation, adverse event prediction and drug selection based on complex patient data, and their intersection with pharmacometabolomic evidence merits brief comment as a secondary and largely prospective application of the framework proposed here. To the authors’ knowledge, and based on the published literature and regulatory documentation reviewed for this Perspective, no clinically deployed prescribing system currently uses validated pharmacometabolomic signatures, as distinct from pharmacogenomic genotype data, as a routine input to prescribing decisions. This is an absence-of-evidence assessment rather than a formal systematic review finding, and it should be read accordingly; the discussion that follows is anticipatory rather than descriptive of existing practice.

If such systems were to incorporate pharmacogenomic, metabolomic, clinical and pharmacovigilance data, they might in principle identify high risk biochemical profiles before harm occurs, which would raise the question of whether failure to act on that information should generate responsibility, and if so, whether responsibility should attach to the clinician, institution, software developer, data supplier, manufacturer or regulator. This Perspective describes this prospective obligation as an algorithmic biochemical duty of care, meaning that any such AI prescribing tool would need to be clinically validated, explainable, auditable and responsive to individualised metabolic risk before it could reasonably ground a legal duty. Black box prediction would be especially problematic where a risk score influences drug selection but cannot be explained to clinicians, patients or courts, a concern consistent with wider debates on predictive analytics, machine learning, accountability, transparency and health data governance (Cohen et al., 2014; Price, 2015; Rajkomar et al., 2019; Topol, 2019; Vayena et al., 2018). The immediate question is not whether such systems can eventually identify biochemical risk, but whether, once they exist in deployable form, their outputs can be clinically justified, legally interrogated and ethically governed.

## Regulatory governance and biochemical privacy

Beyond its primary application to forensic toxicology and criminal responsibility, pharmacometabolomic data raise governance questions as a secondary implication of the framework proposed here, because such data are simultaneously clinical and forensic in character. A metabolomic profile may reveal medication exposure, adherence, disease state, inflammatory status, diet related patterns, substance use and vulnerability to adverse drug response. This sensitivity, concerning what the data reveal about a person’s clinical and behavioural state, is conceptually distinct from personal identifiability. This Perspective does not claim that pharmacometabolomic profiles are currently capable of identifying or re-identifying an individual; the narrower claim is that the information content of such profiles can be clinically and behaviourally sensitive, and that this sensitivity warrants privacy protection independent of any identifiability question, which would itself require dedicated empirical study to resolve. Whether pharmacometabolomic data are, in a given forensic or clinical setting, more revealing of sensitive information than genomic data is likewise an empirical and comparative question that this Perspective does not resolve; no direct comparative evidence is offered here, and the claim should be treated as a hypothesis for future research rather than an established finding.

Regulation should therefore distinguish between clinical use, forensic use and predictive surveillance. Clinical use should be grounded in consent, analytical validity and demonstrable utility. Forensic use should require chain of custody, validated interpretation and judicial gatekeeping. Predictive surveillance should be approached with particular caution because biochemical data of this kind could, in principle, be misused for discrimination, coercive monitoring or unsupported behavioural inference, although the extent of this risk has not been empirically established and is raised here as a governance concern warranting precaution rather than as a documented harm. A robust governance framework must integrate pharmacology, law, ethics, forensic science and data protection rather than treating metabolomic signatures as ordinary laboratory values.

## Limitations

This Perspective has several limitations that should guide its interpretation. First, to the authors’ knowledge, no pharmacometabolomic signature currently validated in the literature has been shown to reliably identify individual level drug related impairment, voluntariness or capacity in a manner that would satisfy clinical or forensic validity; this is an absence-of-evidence assessment based on the literature engaged with in this Perspective rather than a formal systematic review, and the framework proposed here is accordingly prospective rather than descriptive of an available evidentiary tool. Second, jurisdictional variability in criminal responsibility doctrine, negligence standards and rules of evidence means that the arguments developed here, particularly those addressing admissibility, are most directly applicable to United States law and would require separate analysis before extension to other legal systems. Third, confounding remains a substantial obstacle to individual causal attribution, because pre-existing psychiatric or neurological illness, concurrent substance use, situational stress, sleep deprivation and other factors can produce behavioural or cognitive changes that overlap with those attributed to drug exposure, and no current pharmacometabolomic method can reliably exclude these alternative explanations. Fourth, pre-analytical instability, including sample handling, storage conditions, time between exposure and sampling, and matrix effects, can alter metabolomic profiles independently of the underlying biological response, and forensic translation will require standardised protocols that adequately control for these sources of variation before any signature could be relied upon in adjudication. Fifth, the qualitative validation classifications summarised in Table 1 reflect the authors’ synthesis of the literature and argument developed in the corresponding sections of the text rather than a formal, independently graded evidence review, and should be read as an interpretive summary rather than a systematic assessment.

**TABLE 1:** Precision legal FRAMEWORK for pharmacometabolomic evidence.

Domain	Biochemical basis	Legal relevance	Current level of validation	Principal risk
Criminal responsibility	Neuroactive metabolite variability	Assessment of mens rea, capacity and voluntariness	Biological plausibility established; individual-level attribution not yet validated	Biochemical determinism
Forensic toxicology	Individualised metabolic response	Interpretation of impairment and toxicological ambiguity	Used in exploratory or research forensic applications for some purposes; not established for routine forensic use, and behavioural attribution is not validated	Lack of validated thresholds
Medical negligence	Knowable metabolic risk	Evaluation of breach of duty and standard of care	Regulatory recognition exists for pharmacogenomic biomarkers; corresponding pharmacometabolomic regulatory recognition, clinical actionability and standard of care remain undeveloped for most agents	Unclear implementation threshold
Product liability	Vulnerable metabolic subgroups	Assessment of warning adequacy and causation	Conceptual; subgroup-specific causal evidence largely unavailable	Causation uncertainty
AI guided prescribing	Predictive biochemical risk modelling	Algorithmic negligence and accountability	Prospective; no deployed system currently uses validated pharmacometabolomic data for prescribing, to the authors’ knowledge	Opacity of model reasoning
Regulation	Validated biomarker evidence	Admissibility, labelling and oversight	Partial; pharmacogenomic labelling established, pharmacometabolomic labelling largely absent	Fragmented governance
Privacy	Dynamic biochemical information content	Control of sensitive biological information	Conceptual; governance frameworks not yet developed	Discrimination and surveillance

Validation status is described qualitatively because no domain in this table currently possesses a pharmacometabolomic signature validated for individual-level legal attribution; the table summarises the prospective research and governance agenda proposed in this Perspective rather than an available evidentiary standard. Classifications in the ‘Current level of validation’ column (established, partial, conceptual, not validated) reflect the authors’ qualitative synthesis of the literature and argument developed in the corresponding sections of the text, rather than a formal evidence-grading exercise; readers should consult the relevant section for the supporting discussion. An informed consent domain included in an earlier version of this table has been removed, as it is not among the secondary applications defined in the scope of this Perspective and was not substantively developed in the text.

## Research and translational priorities

The next phase of this field should move from conceptual possibility to validation. Research should determine whether specific pharmacometabolomic profiles are reproducibly associated with clinically meaningful neurocognitive impairment, adverse behavioural states or toxicological vulnerability. Studies should prioritise prospective designs, standardised sampling windows, harmonised mass spectrometry or nuclear magnetic resonance protocols, integration with pharmacogenomic phenotypes, and carefully defined behavioural or clinical outcomes. Forensic translation will require interlaboratory reproducibility, reference intervals, quality assurance, chain of custody protocols and reporting standards that express uncertainty clearly.

Clinical translation will require decision support systems that communicate biochemical risk without overwhelming clinicians or patients. Legal translation will require judges and expert witnesses to distinguish between biological plausibility, population association and individual causation, and, as set out earlier in this Perspective, between pharmacogenomic actionability, pharmacokinetic or toxicological confirmation of exposure, and validated pharmacometabolomic evidence of behavioural response. Without this hierarchy, pharmacometabolomic evidence may either be ignored despite its relevance or overvalued as deterministic proof. The scientifically responsible approach is regulated integration guided by the validation pathway described throughout this Perspective, not uncritical adoption.

## Discussion

The principal contribution of this Perspective is the concept of individualised biochemical response evidence, described metaphorically as biochemical legal identity, together with a validation pathway distinguishing biological plausibility, analytical validity, clinical validity, forensic validity and legal admissibility. Its primary focus is forensic toxicology and the assessment of criminal responsibility, where the interpretive gap between drug exposure and behavioural effect is most acute and where existing forensic metabolomic literature already provides a partial foundation for exploratory and research applications, though not for routine forensic use (Castillo-Peinado and Luque de Castro, 2016; Dawidowska et al., 2021; Szeremeta et al., 2021). Medical negligence, product liability, AI guided prescribing, pharmaceutical regulation and biochemical privacy are addressed as secondary implications of this same framework, and each would require its own validation and doctrinal development rather than automatic extension from the forensic toxicology discussion.

Pharmacometabolomic signatures are not merely clinical data; they may, once validated, become legally meaningful evidence of how an individual biochemically experienced a pharmacological exposure. This concept differs from conventional toxicology because it addresses response rather than simple presence, and it differs from genomic evidence because it is dynamic, environmentally responsive and temporally anchored to the event under examination. Nevertheless, biochemical determinism must be avoided. Human conduct cannot be reduced to molecular patterning, and legal responsibility cannot be inferred or denied solely from metabolomic variation. The most defensible position is that validated pharmacometabolomic evidence may, in the future, refine legal reasoning when it is scientifically reliable, temporally connected, clinically plausible and legally relevant. In this model, biochemical response evidence would inform responsibility without displacing agency, proportionality or judicial judgement.

## Conclusion

Pharmacometabolomic signatures represent a Frontier interface between precision medicine and legal accountability, and the argument developed in this Perspective is deliberately prospective rather than a claim that validated evidentiary tools already exist. As drug response science becomes increasingly individualised, legal systems, beginning with forensic toxicology and criminal responsibility, will need to reconsider how biochemical variability might affect causation, foreseeability, impairment, negligence and criminal responsibility, provided that the validation pathway described in this Perspective, from biological plausibility to legal admissibility, is followed rather than assumed. A precision jurisprudence framework, understood as a research and governance agenda rather than a present evidentiary standard, is proposed to guide this transition. The future of drug related legal responsibility may depend not only on whether a substance was present, but on how a specific body transformed that substance into a legally meaningful biochemical event, once the science supporting that inference has been established.

## Data Availability

The original contributions presented in the study are included in the article/supplementary material, further inquiries can be directed to the corresponding author.
